# Whole-Genome Duplication and Purifying Selection Contributes to the Functional Redundancy of Auxin Response Factor (*ARF*) Genes in Foxtail Millet (*Setaria italica* L.)

**DOI:** 10.1155/2021/2590665

**Published:** 2021-08-06

**Authors:** You Chen, Bin Liu, Yujun Zhao, Wenzhe Yu, Weina Si

**Affiliations:** ^1^National Engineering Laboratory of Crop Stress Resistance Breeding, School of Life Sciences, Anhui Agricultural University, Hefei 230036, China; ^2^Precision Medicine Research Laboratory of Anhui Province, Hefei, Anhui 230088, China

## Abstract

Auxin response factors (ARFs) play crucial roles in auxin-mediated response, whereas molecular genetics of *ARF* genes was seldom investigated in *Setaria italica*, an important crop and C_4_ model plant. In the present study, genome-wide evolutionary analysis of *ARFs* was performed in *S. italica*. Twenty-four *SiARF* genes were identified and unevenly distributed on eight of the nine chromosomes in *S. italica*. Duplication mode exploration implied that 13 SiARF proteins were originated from whole-genome duplication and suffered purifying selection. Phylogeny reconstruction of SiARFs by maximum likelihood and neighbor-joining trees revealed SiARFs could be divided into four clades. SiARFs clustered within the same clade shared similar gene structure and protein domain composition, implying functional redundancy. Moreover, amino acid composition of the middle regions was conserved in SiARFs belonged to the same clade. SiARFs were categorized into either activators or repressors according to the enrichment of specific amino acids. Intrinsic disorder was featured in the middle regions of ARF activators. Finally, expression profiles of *SiARFs* under hormone and abiotic stress treatment not only revealed their potential function in stress response but also indicate their functional redundancy. Overall, our results provide insights into evolutionary aspects of *SiARFs* and benefit for further functional characterization.

## 1. Introduction

The phytohormone auxin plays remarkable roles in regulating diverse aspects of plant growth and development, through auxin-responsive signaling cascades and gene expressions [1]. Moreover, auxin/indole acetic acid (Aux/IAA) and auxin response factor (ARF) proteins are key factors to regulate the expression of auxin-response genes [2]. In the absence of auxin, Aux/IAA interacts with ARFs, inhibiting the transcriptional regulation of auxin-responsive genes. Accumulation of auxin could release and activate ARFs. Activity-elevated ARFs would further transcriptionally regulate the expression of auxin-related response genes, such as *small auxin up RNA* (*SAUR*) and *Gretchen Hagen 3* (*GH3*), by combining the TGTCTC auxin response elements (AuxREs) in promoters [3].

Typical ARF proteins are distinguished by an N-terminal DNA-binding domain (DBD), C-terminal Aux/IAA domains that are committed in homo- and heterointeraction, and a variable middle region (MR) [4]. The DBD generally consists of plant-specific B-type domain, which could bind specifically to AuxREs and the auxin_resp domain with unknown function [5]. The MR region determinates whether ARFs are transcriptional activators or repressors [6]. Considering its important roles in auxin regulation system in plant, *ARFs* have drawn dramatically attentions. Researchers have found that *osarf12* mutant plants exhibited premature senescence of leaves and resection of floral organs [7]. Similarly, rice plants with *OsARF12* silenced showed leaf curl, short stature, and reduced viability compared with wild type [8].

Increasing researches have proposed that *ARFs* play roles not only in auxin perception and regulation, but also in the crosstalk of auxin with other phytohormones, such as abscisic acid (ABA), irreplaceable in plant responses to environmental stress [9]. Consistently, many reports suggested that *ARFs* were also in responsive to various abiotic stress, such as drought, salt, and cold [10–13]. *AtARF2* in *Arabidopsis thaliana* was found to be responsive to low potassium stress by phosphorylation modification [14]. *TasiRNA-ARF* was reported to be involved in maintaining the normal morphogenesis of flowers under stress conditions by fine-tuning changes in the expression of floral development and auxin response-related genes in *A. thaliana* [15]. These results elucidate that *ARF* genes played important roles not only in regulating plant growth and development, but also in response to abiotic stress.

Fast development of sequencing technology has provided unprecedented chances and data basis for evolutionary and functional investigation. *ARFs* have been identified and reported in several plants, like *A. thaliana*, *Oryza sativa*, and *Zea mays* [16–19]. However, relevant reports about *ARFs* were seldom found in *Setaria italica*, which is an ideal C_4_ model for genetics and molecular biology research [20]. In the present study, the family members of *ARFs* were explored in *S. italica* genome widely, followed by chromosomal location analysis. Furthermore, duplication modes involved in *SiARF* members and selection pressure underlying the origination of duplicated *SiARF* gene pairs were also investigated. In addition, phylogeny reconstruction of SiARFs was performed in two phylogenetic trees. Domain composition, amino acid composition of MR regions, gene structure, and tissue-specific expression patterns of *SiARFs* were carefully compared among members in different clades. Finally, hormone treatment, as well as abiotic stress including salinity and PEG, was applied to conduct expression profile analysis of *SiARFs*. Above all, our study serves as the first genome-wide evolutionary analysis of *SiARF* genes and underlies the basis of further analysis.

## 2. Materials and Methods

### 2.1. Identification of *ARF* Genes in *S. italica* Genome

By using the ARF HMM profile (ARF_resp, PF06507) [21], the *ARF* sequences of *O. sativa* and *A. thaliana* were used as a query to identify the homologous protein sequences in the foxtail millet protein database (http://www.phytozome.net/BlastP) [22]. The Pfam database (http://Pfam.sanger.ac.uk/) and SMART [23] were used to further confirm each predicted *ARF* gene.

### 2.2. Identifying Protein Characteristics and Mapping *SiARF* Genes on Chromosome

The amino acid (AA) composition, molecular weight (kDa), and theoretical pI (PI) were analyzed by ExPASy (http://web.expasy.org/protparam/). 24 SiARF proteins were accurately distinguished between 8 chromosomes by the MapChart software [24].

### 2.3. Gene Duplication Modes and Collinearity Estimation

MCScanX package was employed to characterize collinearity within *S. italica* genome and gene duplication modes *SiARFs* involved in [25]. MCScanX could implement whole-genome BlastP to identify collinear blocks, referred to collinearity relationship within or between species (*E* value was set up to 1*e*-10). Additionally, MCScanX was applied to trace the evolutionary history of *ARF* gene family expansion and classify tandem and segmental/WGD duplicated *ARF* genes according to their copy number and chromosomal distribution.

### 2.4. Estimation of Synonymous and Nonsynonymous Substitution Rates

SiARF sequences were searched by BlastP against protein sequence of *A. thaliana*, *Z. mays*, and *O. sativa* (http://gramene.org/www.phytozome.net) to account the orthologous homology among *S. italica* and other gramineous plants. The multiple alignment of paralogous and orthologous gene pairs was carried out by Clustalx and submitted to PAL2NAL to calculate Ks and/or Ka value by the codeml program in PAML [26].

### 2.5. Sequence Alignment and Phylogenetic Analysis of *SiARFs*

Multiple sequence alignment was performed using the full-length amino acids of ARFs from *S. italica*, *Z. mays*, *O. sativa*, and *A. thaliana* by MAFFT [27]. TrimAl v1.2 was further employed to remove poorly aligned regions with the parameter of –automated1 [28]. Then, the trimmed alignments were submitted to PhyML to identify the best-fit amino acid substitution model. The best-fit amino acid substitution model was JTT+G (−lnL = 21729.13) [29]. Subsequently, maximum likelihood (ML) phylogenetic tree was constructed in PhyML according to the estimated best-fit mode, and fast approximate likelihood-based measures of branch supports (Shimodaira-Hasegawa approximate likelihood ratio test, SH-aLRT) were applied for branch. To build the neighbor-joining (NJ) phylogenetic tree, the full-length amino acids of SiARFs were aligned by MAFFT and submitted to MEGA 7.0 [30]. The significance of each node bootstrap analysis was carried out using 1000 replicates [31].

### 2.6. Expression Profiling of *SiARF* Genes Using Transcriptome Data

The European Nucleotide Archive provided all Illumina *S. italica* RNA-HiSeq reads for four tissues, namely, spica, stem, leaf, and root [SRX128226 (spica), SRX128225 (stem), SRX128224 (leaf), and SRX128223 (root)] [32]. The RNA-seq data removed low quality reads that were screened by NGS toolkit as standard (http://59.163.192.90 : 8080/ngsqctoolkit/) [33]. The RPKM (reads per kilobase per million) method was used for normalizing the number of reads mapped. According to RPKM values, the heat map demonstrated the specific gene expression in different tissues.

### 2.7. Plant Materials, Growth Conditions, and Stress Treatments

Seeds of *S. italica* were grown in an artificial climate chamber at 28 ± 1°C day/23 ± 1°C night temperature with 70 ± 5% relative humidity and natural sunlight during 21 days (March to April, 2017). For drought stress treatment, seedlings were exposed to 20% polyethylene glycol (PEG 6000) or 100 mM ABA that was initiated on the 21th day of normal growth condition. The different samples were collected after 0, 1, 3, 6, 12, and 24 h treatments, respectively. The 20% PEG (dehydration), salinity, ABA, and indole-3-acetic acid (IAA) treatment were performed that counterfeit in previous studies [34–38]. The samples were frozen in liquid nitrogen and stored at -80°C until RNA isolation. The sample experiments were repeated three times to ensure stability and precision.

### 2.8. RNA Extraction and Expression Profiling Analysis

Total RNA was extracted by using the RNAPlus reagent (Takara, Japan) method, followed by standard manufacturer's instructions [39]. The first cDNA strand was generated using the M-MuLV reverse transcriptase experimental kit (Takara Bio Inc., USA). All gene-specific primers were designed using the Primer Express 3.0 software (Applied Biosystems, USA) with default parameters. The product size ranges from 150 to 250 bp for each *SiARF* gene (Table S2). The PCR reaction condition was performed at 95°C for 10 min, followed by 40 cycles at 95°C for 15 s and 60°C for 1 min. The analysis of melting curve (60 to 95°C after 40 cycles) was done to reduce the experimental error in accordance with the principle of biological repetition [40]. *Actin 2* (PF00022) was used as an internal control. The three replicates were carried out. The statistics was analyzed using the DPS software [41].

### 2.9. Intrinsic Disorder Prediction in the SiARF Proteins

Full-length SiARF amino acid sequences were submitted in DisProt by the PONDR-FIT algorithm [42]. Disordered values were presented as a heat map in R using the gplot package with heat map.2 function.

## 3. Results

### 3.1. Identification, Chromosomal Distribution, and Homology Model Analysis of *ARF* Family in *S. italica*

**Figure 1:**
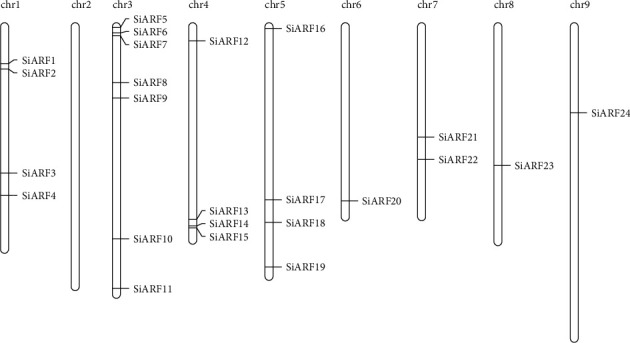
Chromosomal distribution of *SiARFs.*

In the present study, the ARF HMM profile was used to screen the protein database of *S. italica* by the BlastP program. Obtained proteins were further submitted to Pfam website and confirm the existence of ARF and B3 domain. As a result, a total of 24 ARF proteins were found (Table 1). These genes were found to be unevenly distributed on 8 chromosomes, except for chromosome 2 (Figure 1). Chromosome 3 contains seven *SiARFs* (29.1%), and chromosomes 1, 4, and 5 have four *SiARFs*, respectively. Two *SiARFs* were presented on chromosome 7, but only one *SiARF* was found on chromosomes 6, 8, and 9, respectively. Lastly, these *ARF* genes were named according to their chromosomal location from *SiARF01* to *SiARF24* (Table 1).

### 3.2. Duplication and Divergence Rate of the *SiARF* Genes

**Figure 2:**
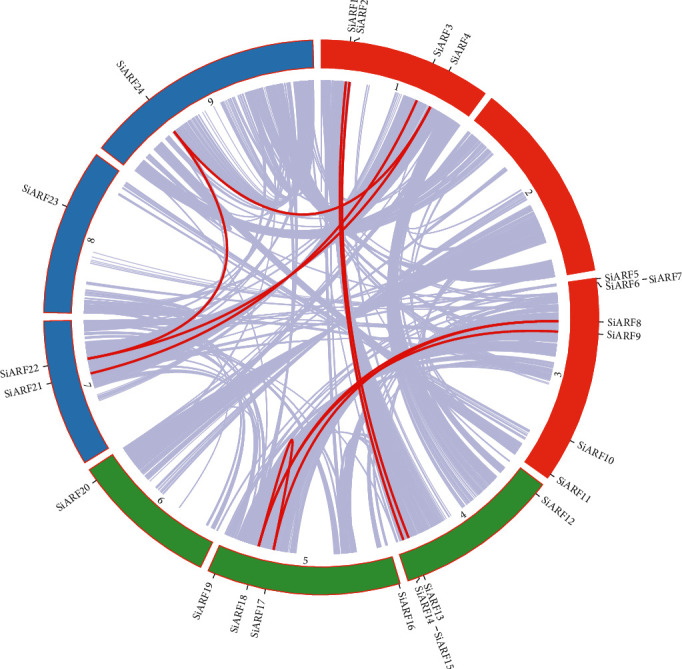
Collinear analysis of *SiARF* genes. Genome-wide collinear genes in *Setaria italica* were marked with grey lines. *SiARF* whole-genome duplication gene pairs were emphasized by red lines.

**Figure 3:**
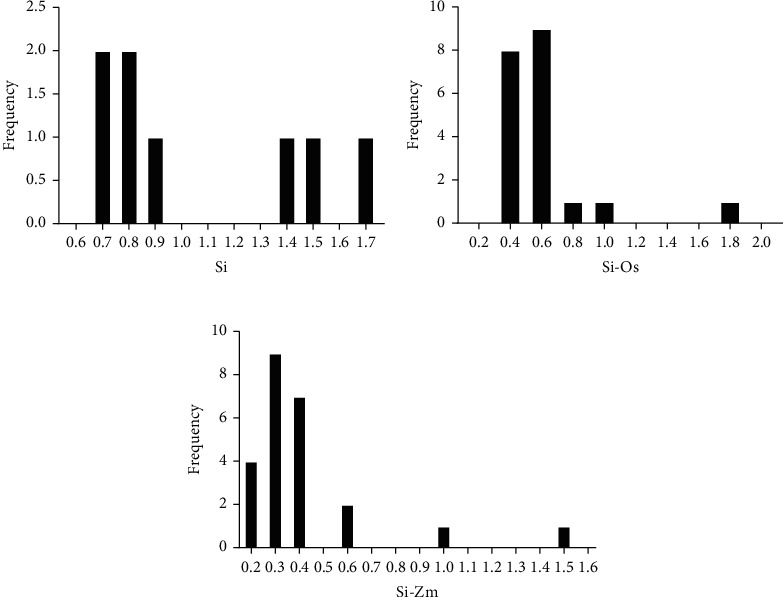
Ks distribution of paralog pairs of *SiARFs.* (a) Orthologous pairs between *S. italica* and *Oryza sativa* and between *S. italica* and *Zea mays*, respectively. The *x*-axis represents the Ks values of paralogous or orthologous gene pairs and was divided into different parts in unit of (a) 0.1 or (c) 0.2. (b) The *y*-axis means the frequency of Ks value relative to each unit. Si represents ARF paralogous in *S. italica*; Si-Os represents ARF orthologous between *S. italica* and *O. sativa*; Si represents ARF orthologous between *S. italica* and *Z. mays*.

Gene duplication, generally related to whole genome/segmental duplication (WGD/SD) and tandem duplication (TD), played important roles in gene family expansion and evolution. Employing blast and MCScanX programs, WGD and TD gene pairs were successfully characterized. Intriguingly, no TD events were found in *SiARFs*, and 13 *SiARFs* were involved in WGD events, including ten WGD gene pairs (Figure 2). To estimate whether selective pressure exits during the evolution and expansion of *SiARFs*, the ratio of nonsynonymous (Ka)/synonymous (Ks) substitution of these WGD pairs was calculated. Generally speaking, the value of Ka/Ks ratio implies selection pressure: Ka/Ks < 1 indicates purifying selection, Ka/Ks = 1 stands for neutral selection, while Ka/Ks > 1 represents positive selection [43]. The maximum value of Ka/Ks was 0.4095, and the minimum value of Ka/Ks was 0.0092 with an average of 0.2129 in *SiARFs* (Table S1). In addition, to roughly deduce the origin of these WGD pairs, the distribution of Ks ratio of paralog in *S. italica* and the ortholog of *S. italica* with *Z. mays* and *O. sativa* was also compared. According our results, the frequency of Ks of paralogs in *S. italica* peaked between 0.8 and 0.9, whereas Ks frequency of ortholog genes between *S. italica* and *Z. mays* or *O. sativa* peaked between 0.6 and 0.8, 0.3, and 0.4, respectively. These results suggest that these WGD paralogs originated before the split of maize and *S. italica* or *S. italica* and *O. sativa* (Figure 3), and their origination time was closer to the split time of *S. italica* and *O. sativa*.

### 3.3. Phylogenetic and Domain Analysis of SiARFs

**Figure 4:**
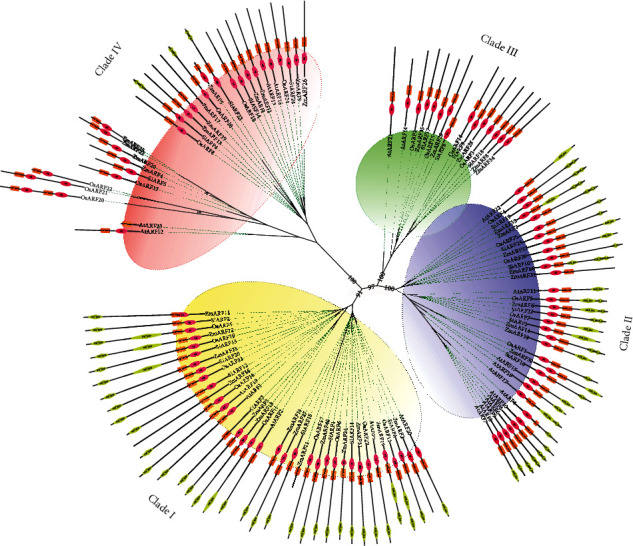
The ML phylogenetic tree constructed by ARF proteins from four plant species. Prefixes in the protein names, including Si, Os, Zm, and At, were used as to denote the species they belong to and represent *S. italica*, *O. sativa*, *Z. mays*, and *Arabidopsis thaliana*, respectively. The phylogenetic tree could be classified into four clades, and each clade was marked with different colors. Yellow, blue, green, and red represented clades I to IV, respectively. Protein domain composition of each ARF members was also exhibited. Red diamonds represented B3 domain. Orange squares represented auxin_resp domain, and yellow diamonds indicated Aux/IAA domain.

To further investigate the evolutionary fate of *SiARF* genes, we constructed an unrooted phylogenetic tree using protein sequences of ARFs from *S. italica*, and those from *O. sativa*, *Z. mays*, and *A. thaliana* were used as outgroups (Figure 4). Totally, 114 ARF members were applied, and a ML phylogenetic tree was successfully constructed. According to the topological structure and bootstrap values of the nodes, the phylogenetic tree could be divided into four clades, namely, clade I (37 members), clade II (33 members), clade III (15 members), and clade IV (29 members). Clade I harbored the most ARF members, while clade III had the least ARF members. Additionally, ARF proteins in *S. italica* were clustered evenly within the three clades, including 9 members in clade I (SiARF1, SiARF2, SiARF6, SiARF7, SiARF11, SiARF12, SiARF13, and SiARF15), 6 in clade II (SiARF3, SiARF10, SiARF16, SiARF19, SiARF21, and SiARF23), 4 SiARF proteins in clade III (SiARF8, SiARF9, SiARF17, and SiARF18), and 5 in clade IV (SiARF4, SiARF5, SiARF14, SiARF22, and SiARF24), respectively (Figure 4). Furthermore, no recent duplication events were detected within SiARF members, while several were organized in maize ARF proteins (Figure 4).

We further examined the protein domain composition of ARFs in these four clades (Figure 4). ARF members, which had closer evolutionary relationship and clustered within the same clade, exhibit conserved protein domain composition. Auxin_resp (PF06507), B3 (PF02362), and Aux/IAA domains are the most conserved domains in surveyed ARF proteins. Almost all the surveyed ARF proteins harbored the conserved auxin_resp (PF06507) and B3 (PF02362) domains. Two ARFs, AtARF23 and ZmARF21, only had the conserved domain auxin_resp, whereas distribution of Aux/IAA domain differed among genes in different clades. ARFs in clades I and II harbored one or two Aux/IAA domains, whereas almost all ARFs in clades III and IV harbored no Aux/IAA domain.

**Figure 5:**
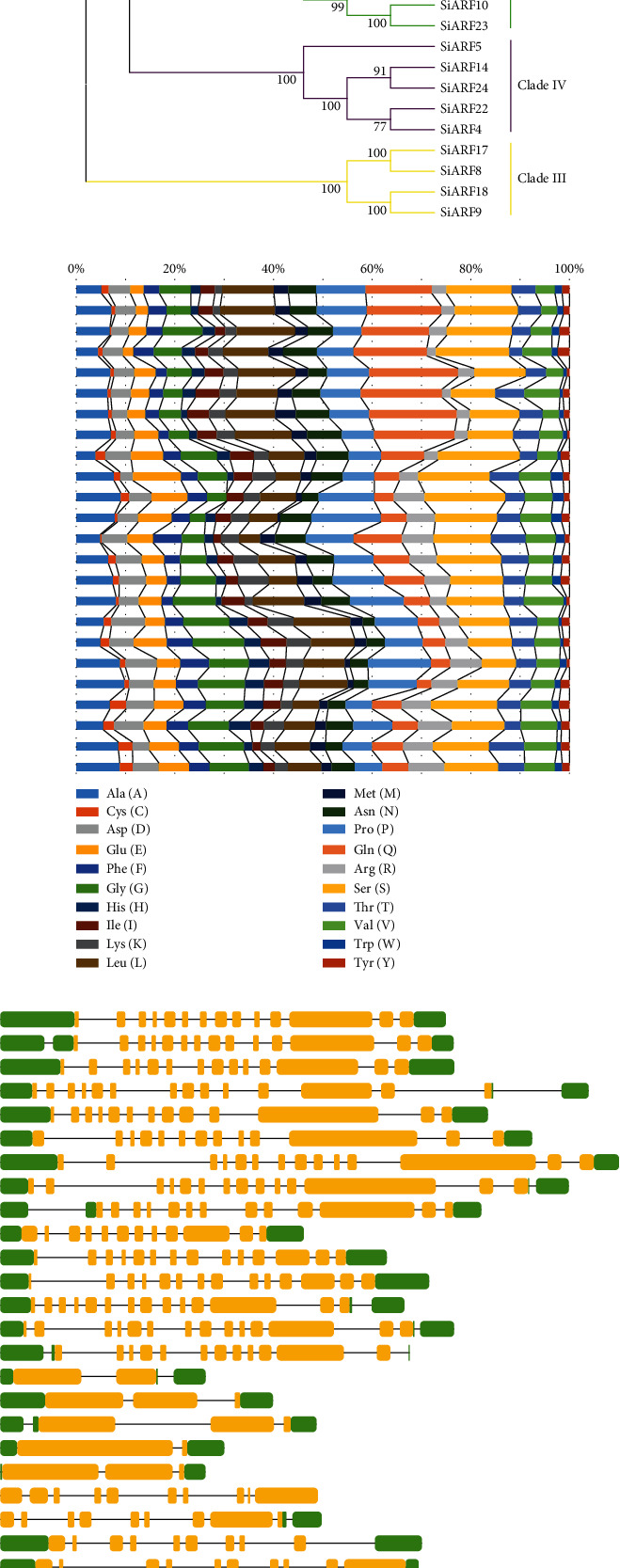
(a) Phylogeny reconstruction and (b) amino acid composition of (c) middle regions and gene structure of *SiARFs.*

To better confirm the accuracy of the phylogeny reconstruction, an NJ tree was built by only SiARF proteins (Figure 5(a)). ARFs in the NJ tree showed the same topological relationships with the MJ tree. Our further analysis mainly focuses on SiARFs employing the NJ tree.

### 3.4. Amino Acid Composition of MR Regions and Gene Structure of *SiARFs*

The protein sequences of MRs between auxin_resp and Aux/IAA are reported to be high variable and associated with the transcriptional activity of *ARF* genes. We subsequently investigated the amino acid composition of MR regions in SiARFs (Figure 5(b)). As expected, SiARFs clustered within the same clades exhibited similar amino acid composition in MR regions. SiARFs in clade I are abundant in S, Q, and L, while SiARFs in clade II are abundant in S and P. In addition, S and G are enriched in the MR regions of SiARFs from clades III and IV.

We further analyzed the gene structure of *SiARF* genes (Figure 5(c)). As expected, the exon-intron structure varies among genes in different clades, while it is relative conserved among genes within in the same clades. Genes in clades I and II had more than 10 exons. Almost all genes in clade I harbored 14 exons, except for *SiARF20* and *SiARF7*, both of which had 13 exons. Five genes, including *SiARF21*, *SiARF3*, *SiARF19*, and *SiARF10* had 14 exons, while the other two genes, *SiARF16* and *SiARF23* have 12 exons. Finally, the five genes in clade IV had only three or two exons.

### 3.5. Tissue-Specific Expression Analysis of *SiARFs*

**Figure 6:**
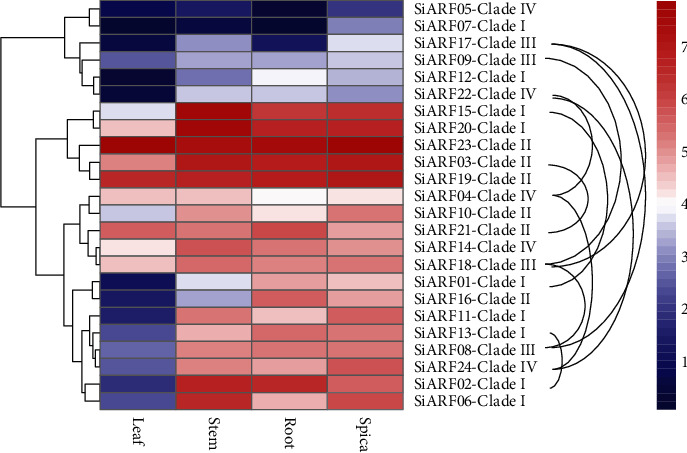
The heat map of the expression level of *SiARFs* in leaf, root, stem, and spica. The color from blue (1) to red (7) represents an increase in the level of gene expression.

Tissue-specific expression profiles of *SiARFs* were estimated by RPKM values, generated from European Nucleotide Archive. The expression pattern of all *SiARF* genes was identified to simulate precise properties through the four tissues: root, stem, leaf, and spica. Results demonstrated the multifarious and dynamics expression profiles of all *SiARFs* in the four surveyed tissues (Figure 6). These 24 *SiARF* genes expressed in at least one tissue (PRKM > 1). *SiARF03*, *SiARF04*, *SiARF19*, *SiARF21*, and *SiARF23* expressed highly in all the four tissues (PRKM > 5). Furthermore, genes in the same clades showed dynamics expression patterns. Genes in clade I had distinct expression levels, while almost all genes in clade II are universally and highly expressed in four surveyed tissues. Remarkably, *SiARF23* exhibited high expression than other *SiARFs* in leaf. In addition, we also identified the expression between gene duplicated pairs. Six of the ten duplicate gene pairs (*SiARF1/15*, *SiARF2/13*, *SiARF22/24*, *SiARF8/17*, *SiARF9/18*, and *SiARF17/18*) resulted from WGD exhibited significant distinct expression profiles in the four surveyed tissues (two-way ANOVA test, *p* < 0.05), while four duplicate gene pairs (*SiARF3/21*, *SiARF4/22*, *SiARF4/24*, and *SiARF8/18*) shared similar expression profiles (two-way ANOVA test, *p* > 0.05), indicating functional redundancy.

### 3.6. Expression Patterns of *SiARFs* under Hormone and Abiotic Stress Treatments

**Figure 7:**
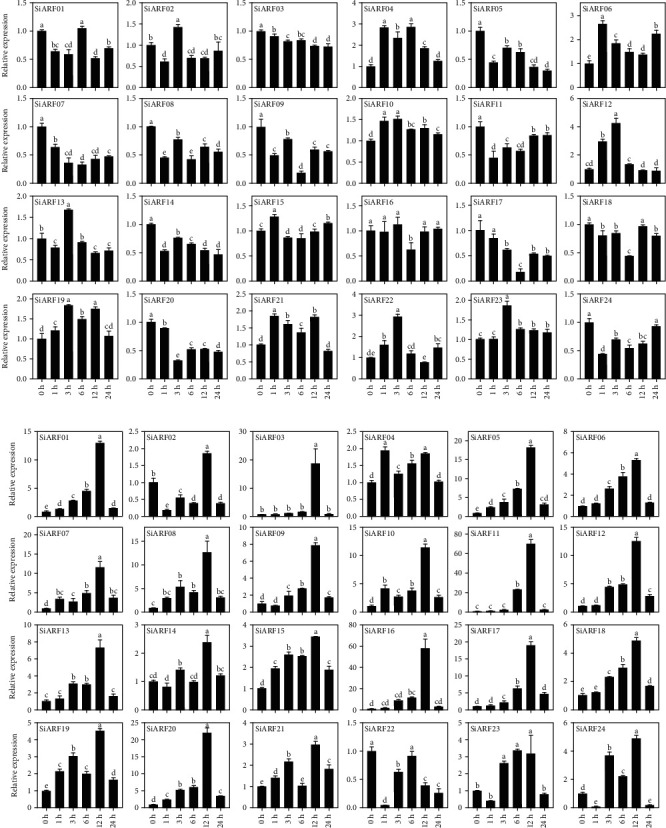
The relative expression of 24 *SiARFs* analyzed using real-time quantitative PCR under (a) indole-3′-acetic acid (IAA) treatment and (b) abscisic acid (ABA) treatment after 0 h, 1 h, 3 h, 6 h, 12 h, and 24 h. All the experimental results were based on three biologic replicates. Error bars imply ±SD for three biological replicates. Different letters indicate significant differences (*p* < 0.05).

It is generally known that the regulation pathway of auxin and the expression level of *ARF* genes have an important correlation. The induction treatment of exogenous auxin was beneficial to the expression of *ARF* family gene in plant. Previous experimental results reveal that *SiARF* members have complex biological effects on the secretion of auxin. In the present study, under the treatment of 1 *μ*m IAA, the real-time quantitative PCR analysis showed that huge variations exist in all *SiARF* genes (Figure 7(a)). For example, in IAA treatment condition, the expression of almost half of all *SiARF* genes (*SiARF03*, *SiARF05*, *SiARF07*, *SiARF08*, *SiARF09*, *SiARF11*, *SiARF14*, *SiARF17*, *SiARF18*, *SiARF20*, *SiARF20*, and *SiARF24*) were subdued continually. Nevertheless, 8 *ARF* genes (*SiARF04*, *SiARF06*, *SiARF10*, *SiARF12*, *SiARF19*, *SiARF21*, *SiARF22*, and *SiARF23*) were upregulated by exogenous IAA treatment. The remaining *ARF* gene expression levels show irregular expression profiles.

To further investigate whether the *SiARFs* were involved in ABA signaling pathway, seedlings were used to be disposed ABA. The results showed that almost all *SiARFs* were constantly upregulated by ABA stimuli until at 12 h (Figure 7(b)), besides *SiARF04* expressed highest at 1 h. In addition to this, the expression level of *SiARF22* showed a downregulation tendency. Comparing the relative expression of the *SiARF* family in ABA stimuli, It is not hard to find that *SiARF11* was evidenced to superlatively expressed (>70-fold) than others. In summary, *SiARF* genes had largely response effect on plant hormones in theory.

**Figure 8:**
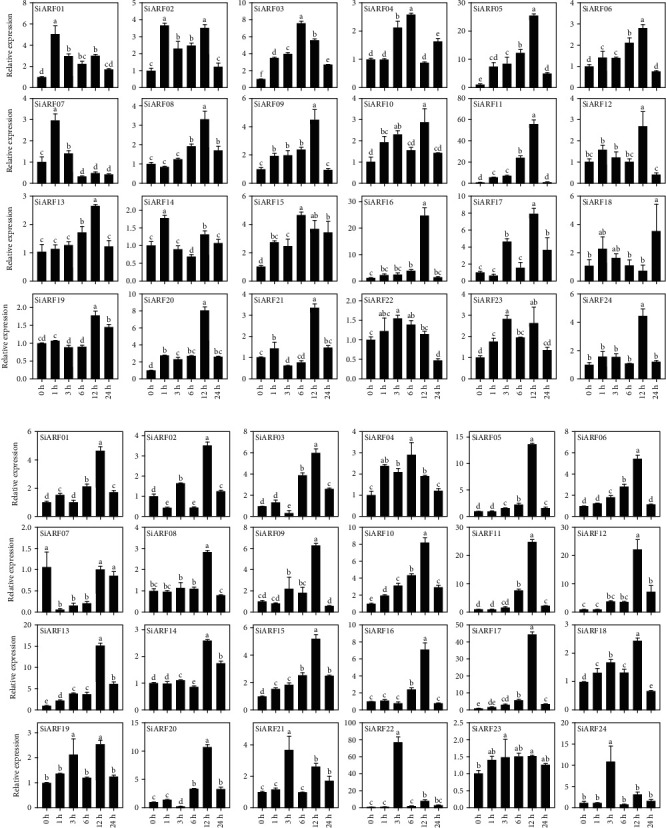
The relative expression of 24 *SiARFs* analyzed using real-time quantitative PCR under (a) PEG stress treatment and (b) salinity stress treatment after 0 h, 1 h, 3 h, 6 h, 12 h, and 24 h. All the experimental results were based on three biologic replicates. Error bars imply ±SD for three biological replicates. Different letters indicate significant differences (*p* < 0.05).

To investigate the response mechanism of *SiARF* transcription family members, the real-time quantitative PCR was used to analyze the potential functions of *SiARFs* during abiotic stress (salinity and dehydration). Firstly, during the different period (1 h, 3 h, 6 h, 12 h, and 24 h) duration of PEG treatment, the results were visualized by histogram. Interestingly, we found that all *SiARF* genes were upregulated at the bulk of time points (Figure 8(a)). The quantitative expression of all *SiARFs* found that *SiARF01*, *SiARF02*, and *SiARF14* peaked at 1 h, and *SiARF03*, *SiARF04*, and *SiARF15* were highest expressed after 6 h. The expression of *SiARF22* and *SiARF23* was increased until at 3 h, and *SiARF07* and *SiARF18* peaked at 24 h. In addition, the transcription level of *SiARF05*, *SiARF06*, *SiARF08*, *SiARF09*, *SiARF10*, *SiARF11*, *SiARF12*, *SiARF13*, *SiARF16*, *SiARF17*, *SiARF19*, *SiARF20*, *SiARF21*, and *SiARF24* reached the expression peak at 12 h; subsequently, the expression levels were reduced. Contrarily, the relative expression of *SiARF11* steadily increased more than 50-fold at 12 h.

Salinity stress changes the permeability of plant cells to affect the growth and development of plants by osmotic stress and the ion toxicity. Hence, we also designed salinity treatment expression profiles to get insight into potential tolerance effects of foxtail millet *ARF* genes. The results found that all *SiARF* genes have incurred variations in different treatment phases (Figure 8(b)). It was observed that all *SiARF* genes appear transcriptional level increased in different time points. Strikingly, only *SiARF04* was accompanied by constant expression until 6 h reaches maximum value, and three genes (*SiARF21*, *SiARF22*, and *SiARF24*) showed highest expression at 3 h. However, all of other genes (*SiARF01*, *SiARF02*, *SiARF03*, *SiARF04*, *SiARF05*, *SiARF06*, *SiARF07*, *SiARF08*, *SiARF09*, *SiARF10*, *SiARF11*, *SiARF12*, *SiARF13*, *SiARF14*, *SiARF15*, *SiARF16*, *SiARF17*, *SiARF18*, *SiARF19*, *SiARF20*, and *SiARF23*) were measured that all were accidentally upregulated in treatment time and peaked at 12 h. Noteworthy, five genes (*SiARF05*, *SiARF11*, *SiARF17*, *SiARF22*, and *SiARF24*) appeared the idiocratic higher expression at the time point than other treatment duration. The *SiARF22* was even upregulated more than 70-fold at 3 h. Data results indicate that *SiARFs* play an important role in regulating the dehydration stress and salinity stress.

## 4. Discussion

*ARF* genes are key factors in the auxin-induced signaling cascades and gene expression, regulating all aspects of plant metabolism progress and participating in plant stress response [14]. *ARF* families in plant species have multiple gene members, which, to a certain extent, could explain why simple auxin-to-ARF pathway could manipulate such many aspects of physiological metabolism [2]. A broad exploration to the evolutionary patterns and phylogenic relationship between *ARFs* could benefit for deciphering the functional specialization in *ARFs*. Hence, in this study, a genome-wide survey of *ARF* genes in *S. italica* was presented.

In *S. italica*, twenty-four *SiARF* genes were identified, number of which was similar to that in *A. thaliana* (23) and *O. sativa* (25), but less than that in *Z. mays* (36) [16, 17, 19]. Gene duplication, mainly including WGD and TD, was generally believed to involve in gene expansion of plant multiple gene families. Moreover, genes generated by different duplication modes had different functional fates. WGD duplicates were supposed to contribute to functional redundancy, while TD duplicates could cope with rapidly changing environments and gain functional novelty [44]. In the present study, 13 out of 24 *SiARFs* were deduced to experience WGD events and 10 WGD gene pairs were identified (Figure 2). We also found evolutionary force underlying WGD gene pairs was purifying selection. Ka distribution implied the ancient origin of *SiARF* pairs, which were originated before the split of *S. italica* with *O. sativa* or with *Z. mays*. Considering broad impact of auxin-induced signaling cascades and the upstream roles of *ARFs*, these WGD pairs may contribute to the function redundancy or specification.

Phylogenetic analysis of *SiARFs* was also performed. Firstly, an ML tree was constructed, applying ARFs from other three genes as outgroups (Figure 4). In the ML tree, ARFs could apparently be classified into four clades. Moreover, genes within the same clades shared similar protein domain composition. We also constructed an NJ tree with only SiARFs (Figure 5). As expected, SiARFs in the NJ tree exhibited almost the same phylogenic relationship with those in the ML tree. The consistence between the NJ and ML trees confirmed the accuracy and repeatability of the phylogeny reconstruction. With restriction to SiARFs, proteins in clade II and clade I harbored integrated functional domain, including B3, Aux_resp, and Aux/IAA domains, while majority of the proteins in clade III and clade IV lacked Aux/IAA domain. Considering the homo- or heterointeraction of Aux/IAA domain, proteins in clades III and IV may function as monomers. In addition, the amino acid composition of MR regions was reported to determine whether an ARF protein is a transcriptional activator or repressor [3]. In previous advances, Q, S, and L are enriched in the MR regions of ARF activators. Other ARFs without Q-rich MR regions are almost probably transcriptional repressors. In our results, MR regions of SiARFs in clade I are enriched in Q, S, and L (Figure 5(b)). Furthermore, those OsARFs in clade I had been experimentally confirmed to be transcriptional activators and interact with OsAux/IAA proteins [6]. These outcomes strongly imply that SiARFs in clade I are transcriptional activators and could interact with SiAux/IAA. SiARFs in clades II to IV are most likely to be transcriptional repressors (Figure S2). Moreover, previous advances predicted the presence of intrinsic disorder (ID) in the MR of ARF activators [2]. ID was characterized by lack of 3D structure and enriched with charged and polar amino acid residues. Our results proved that intrinsic disorder was conserved character in the clade I SiARFs (Figure S2), consistent with those in previous research [2]. ID contributes to protein-protein interaction, conditional DNA binding, and posttranslational modifications, through specific and rapid conformational changes [45]. As majority research were concerned on the conserved B3, Aux_resp, and Aux/IAA domains, the discovery of ID could provide new aspect of auxin output control through *ARF*-related pathway.

It is well known that *ARFs* play central in auxin-related pathway. Concentration of auxin could regulate the activity of *ARFs* [3]. In our results, *SiARFs* are responsive to exogenous IAA treatment, which may be owing to that exogenous IAA could influence the homeostasis of auxin (Figure 7(a)). Additionally, alteration of the concentration of auxin could also induce the ABA-related pathway [46]. Moreover, ABA is another important hormone and involves in response to diverse stress [47]. In previous researches, *ARFs* were believed to be promising candidates involving in environmental stress and hormone signaling. *IbARF5* from sweet potato could significantly improve the tolerance to salt and drought in transgenic *A. thaliana*, associated with ABA biosynthesis [48]. *ARF4* in poplar inhibited auxin signaling in lateral root (LR) formation under salt stress [49]. In the present study, we found that almost all *SiARFs* were upregulated in leaves by exogenous ABA. Subsequently with abiotic stress treatment (NaCl and PEG), majority of *SiARFs* were also found responsive, exhibiting either upregulated or downregulated expression tendency (Figures 7(b) and 8). These findings strongly imply that *SiARFs* are associated with abiotic stress, in an ABA-dependent pathway, showing the functional conversation or redundancy. Our results could provide some bases for further functional characterization, especially those related to stress and hormone signaling.

## 5. Conclusions

*ARFs* are key output control of auxin-related pathway. In the present study, evolutionary patterns of *ARFs* genes in *S. italica* were genome widely performed. 13 out of the 24 SiARF proteins were originated from whole-genome duplication, suffering purifying selection. Phylogeny reconstruction of *SiARFs* by ML and NJ trees revealed that *SiARFs* could be categorized into four clades. *SiARFs* clustered within the same clade shared similar gene structure and protein domain composition, implying functional redundancy. Moreover, amino acid composition of the middle regions (MR) was conserved in *SiARFs* belonged to the same clade. *SiARFs* in clade I are suggested to be transcriptional activators and may interact with SiIAA/Aux, while *SiARFs* in clades II to IV are most likely to be transcriptional repressors. Intrinsic disorder was featured in the MR regions of *ARF* activators. In addition, various *SiARFs* are induced or repressed under hormone and abiotic stress treatments, which revealed their potential function in stress response and functional redundancy. Overall, the present study provides a “mugshot” of *SiARFs* and provides a theoretical basis for further functional and molecular mechanism analysis.

## Figures and Tables

**Table 1:** The characteristics of identified *auxin response factor* gene family in *Setaria italica*.

Gene name^a^	Locus	Chr location	AA^b^	PI^c^	MW (kDa)^d^
*SiARF1*	Seita.1G077200	1 : 6988237…6994473	907	5.45	99.6988
*SiARF2*	Seita.1G090900	1 : 8014277…8022232	1133	5.9	125.4214
*SiARF3*	Seita.1G195500	1 : 27687425…27693427	673	5.76	74.9183
*SiARF4*	Seita.1G241500	1 : 31927733…31930605	696	6.86	74.5453
*SiARF5*	Seita.3G003300	3 : 165345…168218	502	5.75	54.4367
*SiARF6*	Seita.3G020000	3 : 1162915…1171149	835	6.3	92.7573
*SiARF7*	Seita.3G028100	3 : 1711500…1718231	937	5.79	103.1312
*SiARF8*	Seita.3G147000	3 : 10559752…10564250	577	9.14	64.2633
*SiARF9*	Seita.3G179300	3 : 13481507…13487361	579	8.33	64.254
*SiARF10*	Seita.3G321500	3 : 40156717…40163068	841	6.25	92.7379
*SiARF11*	Seita.3G394000	3 : 49527955…49534308	897	5.66	98.8577
*SiARF12*	Seita.4G040100	4 : 2680871…2687694	1054	6.09	116.3922
*SiARF13*	Seita.4G240600	4 : 36454278…36462936	1084	6.12	120.7337
*SiARF14*	Seita.4G257800	4 : 37696236…37700051	686	7.05	74.697
*SiARF15*	Seita.4G262300	4 : 38057919…38064263	931	5.95	102.7815
*SiARF16*	Seita.5G004300	5 : 322788…327036	687	5.59	76.8788
*SiARF17*	Seita.5G265200	5 : 32737893…32742338	667	6.42	73.7489
*SiARF18*	Seita.5G321300	5 : 37047017…37052916	685	6.84	74.7693
*SiARF19*	Seita.5G438100	5 : 45465923…45471577	809	6.01	90.7756
*SiARF20*	Seita.6G212000	6 : 32960180…32967624	1099	6.13	121.6518
*SiARF21*	Seita.7G108100	7 : 20883066…20888476	663	5.59	73.4238
*SiARF22*	Seita.7G169600	7 : 25114768…25117902	677	8.05	72.674
*SiARF23*	Seita.8G135700	8 : 26264640…26270855	811	6.6	90.0333
*SiARF24*	Seita.9G219800	9 : 16301628…16306053	684	6.71	74.705

^a^The name of a gene named according to its order on the chromosome. ^b^Protein sequence length. ^c^Isoelectric point. ^d^Molecular weight.

## Data Availability

Our research data has been submitted as supplementary materials.

## Supplementary Materials

Supplementary Materials See Figure S1-S2 and Table S1-S3 in the Supplementary Material 1. Figure S1: the ML phylogenetic tree constructed by ARF proteins from four plant species. Figure S2: intrinsic disorder and transcriptional activity prediction of SiARFs. Green indicated unstructured protein scores. Table S1: synonymous and nonsynonymous values of *SiARF* WGD duplicate pairs. Table S2: synonymous values of orthologous *ARF* gene pairs between *S. italica* and *O. sativa* and between *S. italica* and *Z. mays*. Table S3: primers used in the real-time quantitative PCR assay. See coding sequencing sequences of *ARFs* of four surveyed species in the Supplementary Material 2. See full-length amino acid sequences of ARFs of four surveyed species in the Supplementary Material 3.

## References

[1] Li S.-B., Xie Z.-Z., Hu C.-G., Zhang J.-Z. (2016). A review of auxin response factors (ARFs) in plants. *Frontiers in Plant Science*.

[2] Roosjen M., Paque S., Weijers D. (2018). Auxin response factors: output control in auxin biology. *Journal of Experimental Botany*.

[3] Tiwari S. B., Hagen G., Guilfoyle T. (2003). The roles of auxin response factor domains in auxin-responsive transcription. *Plant Cell*.

[4] Song S., Hao L., Zhao P. (2019). Genome-wide Identification, Expression Profiling and Evolutionary Analysis of Auxin Response Factor Gene Family in Potato (*Solanum tuberosum* Group Phureja). *Scientific Reports*.

[5] Boer D. R., Freire-Rios A., van den Berg W. A. M. (2014). Structural basis for DNA binding specificity by the auxin-dependent ARF transcription factors. *Cell*.

[6] Shen C., Wang S., Bai Y. (2010). Functional analysis of the structural domain of ARF proteins in rice (*Oryza sativa* L.). *Journal of Experimental Botany*.

[7] Wang S. K., Zhang S. N., Sun C. D. (2014). Auxin response factor (OsARF12), a novel regulator for phosphate homeostasis in rice (*Oryza sativa*). *The New Phytologist*.

[8] Attia K. A., Abdelkhalik A. F., Ammar M. H. (2009). Antisense phenotypes reveal a functional expression of OsARF1, an auxin response factor, in transgenic rice. *Current Issues in Molecular Biology*.

[9] Finkelstein R. R., Gampala S. S., Rock C. D. (2002). Abscisic acid signaling in seeds and seedlings. *Plant Cell*.

[10] Jain M., Khurana J. P. (2009). Transcript profiling reveals diverse roles of auxin-responsive genes during reproductive development and abiotic stress in rice. *The FEBS Journal*.

[11] Wang S., Bai Y., Shen C. (2010). Auxin-related gene families in abiotic stress response in *Sorghum bicolor*. *Functional & Integrative Genomics*.

[12] Ha C. V., le D. T., Nishiyama R. (2013). The auxin response factor transcription factor family in soybean: genome-wide identification and expression analyses during development and water stress. *DNA Research*.

[13] Sekhwal M. K., Swami A. K., Sharma V., Sarin R. (2015). Identification of drought-induced transcription factors in Sorghum bicolor using GO term semantic similarity. *Cellular & Molecular Biology Letters*.

[14] Zhao S., Zhang M. L., Ma T. L., Wang Y. (2016). Phosphorylation of ARF2 relieves its repression of transcription of the K+ transporter gene HAK5 in response to low potassium stress. *Plant Cell*.

[15] Matsui A., Mizunashi K., Tanaka M. (2014). tasiRNA-ARF pathway moderates floral architecture in Arabidopsis plants subjected to drought stress. *BioMed Research International*.

[16] Okushima Y., Overvoorde P. J., Arima K. (2005). Functional genomic analysis of the *auxin response factor* gene family members in Arabidopsis thaliana: unique and overlapping functions of *ARF7* and *ARF19*. *Plant Cell*.

[17] Wang D. K., Pei K. M., Fu Y. P. (2007). Genome-wide analysis of the *auxin response factors* (ARF) gene family in rice (*Oryza sativa*). *Gene*.

[18] Jiang H. Y. (2010). Genome-wide analysis and evolution of the auxin response factor (ARF) gene family in *Sorghum bicolor*. *Journal of Anhui Agricultural University*.

[19] Liu Y., Jiang H. Y., Chen W. J. (2011). Genome-wide analysis of the auxin response factor (ARF) gene family in maize (*Zea mays*). *Plant Growth Regulation*.

[20] Li P., Brutnell T. P. (2011). Setaria viridis and Setaria italica, model genetic systems for the Panicoid grasses. *Journal of Experimental Botany*.

[21] Bateman A., Coin L., Durbin R. (2004). The Pfam protein families database. *Nucleic Acids Research*.

[22] Goodstein D. M., Shu S. Q., Howson R. (2012). Phytozome: a comparative platform for green plant genomics. *Nucleic Acids Research*.

[23] Letunic I., Khedkar S., Bork P. (2021). SMART: recent updates, new developments and status in 2020. *Nucleic Acids Research*.

[24] Cheng Y., Li X. Y., Jiang H. Y. (2012). Systematic analysis and comparison of nucleotide-binding site disease resistance genes in maize. *The FEBS Journal*.

[25] Wang Y. P., Tang H. B., DeBarry J. D. (2012). MCScanX: a toolkit for detection and evolutionary analysis of gene synteny and collinearity. *Nucleic Acids Research*.

[26] Suyama M., Torrents D., Bork P. (2006). PAL2NAL: robust conversion of protein sequence alignments into the corresponding codon alignments. *Nucleic Acids Research*.

[27] Katoh K., Rozewicki J., Yamada K. D. (2019). MAFFT online service: multiple sequence alignment, interactive sequence choice and visualization. *Briefings in Bioinformatics*.

[28] Capella-Gutierrez S., Silla-Martinez J. M., Gabaldon T. (2009). trimAl: a tool for automated alignment trimming in large-scale phylogenetic analyses. *Bioinformatics*.

[29] Guindon S., Dufayard J. F., Lefort V., Anisimova M., Hordijk W., Gascuel O. (2010). New algorithms and methods to estimate maximum-likelihood phylogenies: assessing the performance of PhyML 3.0. *Systematic Biology*.

[30] Kumar S., Stecher G., Tamura K. (2016). MEGA7: molecular evolutionary genetics analysis version 7.0 for bigger datasets. *Molecular Biology and Evolution*.

[31] Tamura K., Dudley J., Nei M., Kumar S. (2007). MEGA4: molecular evolutionary genetics analysis (MEGA) software version 4.0. *Molecular Biology and Evolution*.

[32] Cochrane G., Alako B., Amid C. (2013). Facing growth in the European Nucleotide Archive. *Nucleic Acids Research*.

[33] Patel R. K., Jain M. (2012). NGS QC toolkit: a toolkit for quality control of next generation sequencing data. *PLoS One*.

[34] Xiang Y., Huang Y. M., Xiong L. Z. (2007). Characterization of stress-responsive *CIPK* genes in rice for stress tolerance improvement. *Plant Physiology*.

[35] Lu G., Gao C. X., Zheng X. N., Han B. (2009). Identification of *OsbZIP72* as a positive regulator of ABA response and drought tolerance in rice. *Planta*.

[36] Zhao Y., Zhou Y. Q., Jiang H. Y. (2011). Systematic analysis of sequences and expression patterns of drought-responsive members of the *HD-Zip* gene family in maize. *PLoS One*.

[37] Mishra A. K., Muthamilarasan M., Khan Y., Parida S. K., Prasad M. (2014). Genome-wide investigation and expression analyses of WD40 protein family in the model plant foxtail millet (*Setaria italica* L.). *PLoS One*.

[38] Li S. B., OuYang W. Z., Hou X. J., Xie L. L., Hu C. G., Zhang J. Z. (2015). Genome-wide identification, isolation and expression analysis of *auxin response factor* (*ARF*) gene family in sweet orange (Citrus sinensis). *Frontiers in Plant Science*.

[39] Logemann J., Schell J., Willmitzer L. (1987). Improved method for the isolation of RNA from plant tissues. *Analytical Biochemistry*.

[40] Kumar K., Muthamilarasan M., Prasad M. (2013). Reference genes for quantitative real-time PCR analysis in the model plant foxtail millet (Setaria italica L.) subjected to abiotic stress conditions. *Plant Cell, Tissue and Organ Culture (PCTOC)*.

[41] Tang Q. Y., Zhang C. X. (2013). Data Processing System (DPS) software with experimental design, statistical analysis and data mining developed for use in entomological research. *Insect Science*.

[42] Xue B., Dunbrack R. L., Williams R. W., Dunker A. K., Uversky V. N. (2010). PONDR-FIT: a meta-predictor of intrinsically disordered amino acids. *Biochimica et Biophysica Acta*.

[43] Yang Z., Gu S., Wang X., Li W., Tang Z., Xu C. (2008). Molecular evolution of the *CPP-like* gene family in plants: insights from comparative genomics of Arabidopsis and rice. *Journal of Molecular Evolution*.

[44] Qiao X., Li Q., Yin H. (2019). Gene duplication and evolution in recurring polyploidization-diploidization cycles in plants. *Genome Biology*.

[45] Liu J. G., Perumal N. B., Oldfield C. J., Su E. W., Uversky V. N., Dunker A. K. (2006). Intrinsic disorder in transcription factors. *Biochemistry-Us*.

[46] Rock C. D., Sun X. (2005). Crosstalk between ABA and auxin signaling pathways in roots of *Arabidopsis thaliana* (L.) Heynh. *Planta*.

[47] Raghavendra A. S., Gonugunta V. K., Christmann A., Grill E. (2010). ABA perception and signalling. *Trends in Plant Science*.

[48] Kang C., He S., Zhai H., Li R., Zhao N., Liu Q. (2018). A sweetpotato *auxin response factor* gene (IbARF5) is involved in carotenoid biosynthesis and salt and drought tolerance in transgenic Arabidopsis. *Frontiers in Plant Science*.

[49] He F., Xu C. Z., Fu X. K. (2018). TheMicroRNA390/TRANS-ACTING short interfering RNA3Module mediates lateral root growth under salt stress via the auxin pathway. *Plant Physiology*.

