# Supplementary material for: Whole-Genome Duplication and Purifying Selection Contributes to the Functional Redundancy of Auxin Response Factor (ARF) Genes in Foxtail Millet (Setaria italica L.)
Source: Int J Genomics. 2021 Aug 6;2021:2590665. doi: 10.1155/2021/2590665 (PMC8369178; doi:10.1155/2021/2590665)
Supplement: Supplementary Materials — See Figure S1-S2 and Table S1-S3 in the Supplementary Material 1. Figure S1: the ML phylogenetic tree constructed by ARF proteins from four plant species. Figure S2: intrinsic disorder and transcriptional activity prediction of SiARFs. Green indicated unstructured protein scores. Table S1: synonymous and nonsynonymous values of SiARF WGD duplicate pairs. Table S2: synonymous values of orthologous ARF gene pairs between S. italica and O. sativa and between S. italica and Z. mays. Table S3: primers used in the real-time quantitative PCR assay. See coding sequencing sequences of ARFs of four surveyed species in the Supplementary Material 2. See full-length amino acid sequences of ARFs of four surveyed species in the Supplementary Material 3. [file 2590665.f1.zip › Supplementary Material 1.pdf]

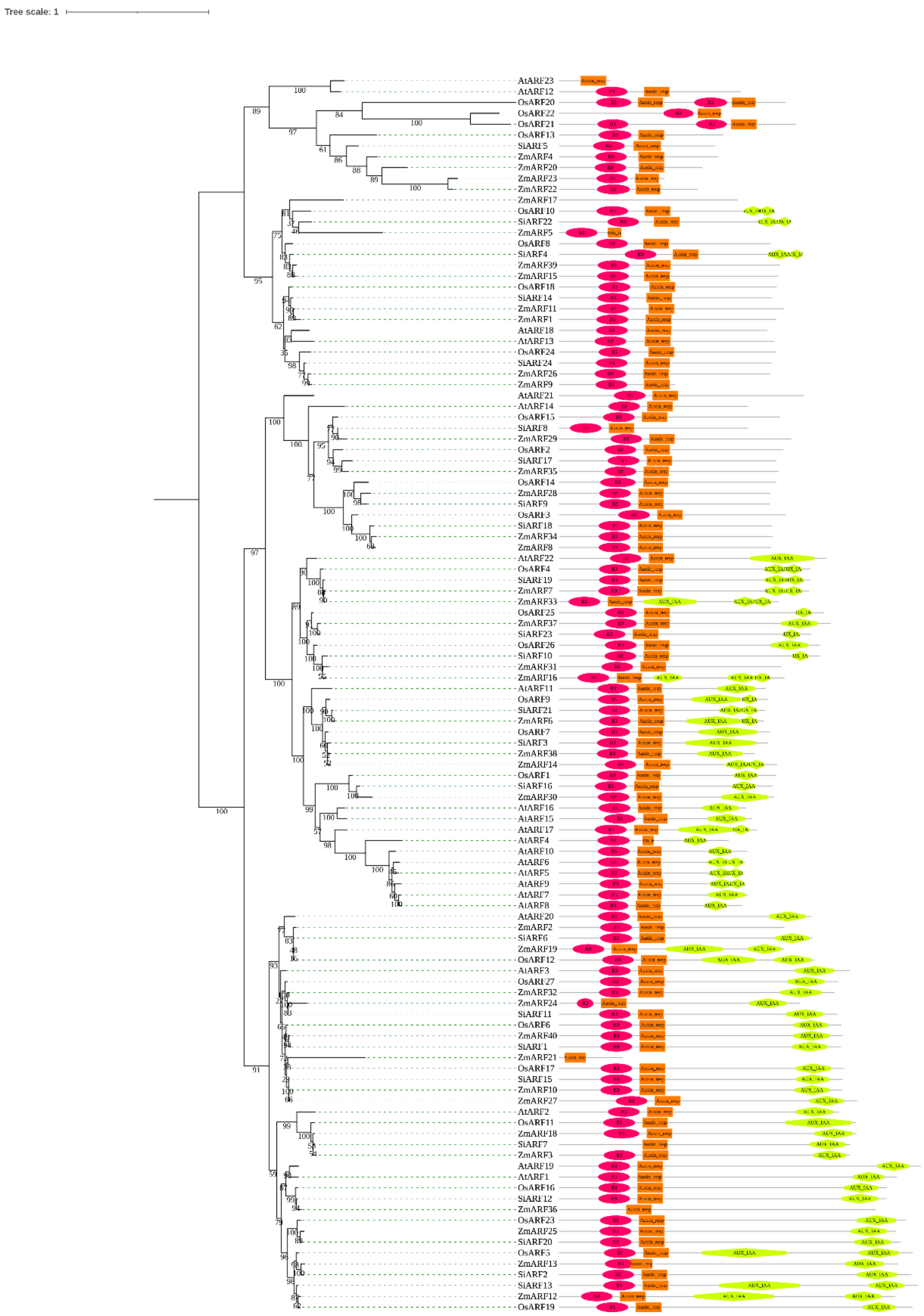

Figure S1: The ML phylogenetic tree constructed by ARF proteins from four plant species. Prefixes in the protein names, including Si, Os, Zm, and At, were used as to denote the species they belong to and represent *S.italica*, *O.sativa*, *Z.mays* and *Arabidopsis thaliana*, respectively. The phylogenetic tree could be classified into four clades and each clades were marked with different colors. Yellow, blue, green and red represented Clade I to IV, respectively. Protein domain composition of each *ARF* members were also exhibited. Red diamonds represented B3 domain. Orange squares represented Auxin_rep domain and yellow diamonds indicated Aux/IAA domain.

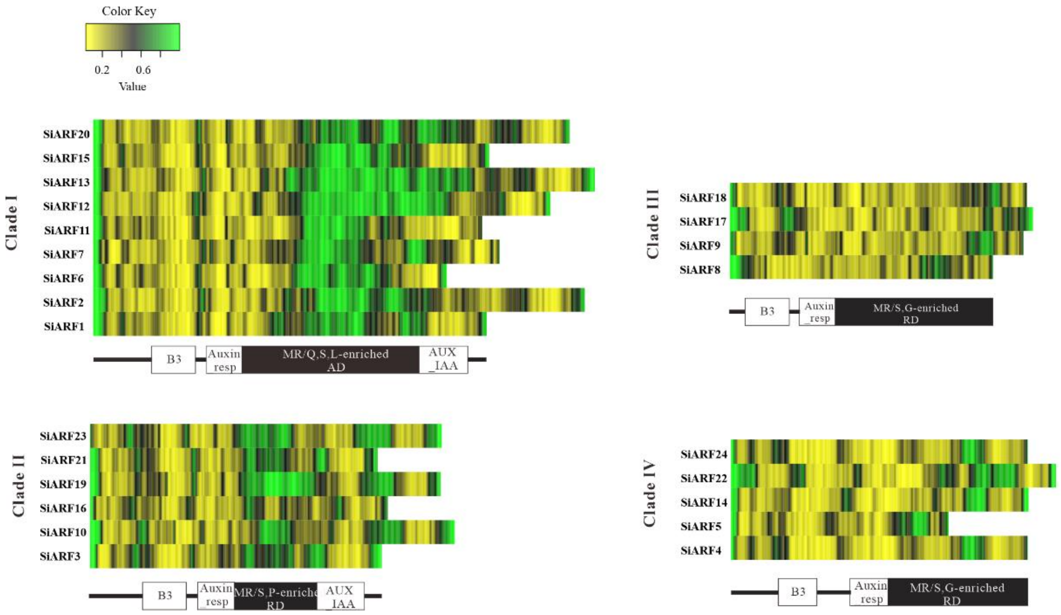

Figure S2: Intrinsic disorder and transcriptionally activity prediction of *SiARFs*. Green indicated unstructured protein scores.

Table S1 Synonymous and non-synonymous values of SiARFs WGD duplicate pairs

Duplicated pairs		Ks	Ka	Ka/Ks
Seita.1G077200.1	Seita.4G262300.1	0.6948	0.1175	0.17
Seita.1G090900.1	Seita.4G240600.1	0.9073	0.1397	0.15
Seita.1G195500.1	Seita.7G108100.1	0.8343	0.1443	0.17
Seita.1G241500.1	Seita.7G169600.1	1.4384	0.2246	0.16
Seita.1G241500.1	Seita.9G219800.1	28.1995	0.2581	0.0092
Seita.7G169600.1	Seita.9G219800.1	35.4072	0.3387	0.0096
Seita.3G147000.1	Seita.5G321300.1	1.6641	0.5038	0.3027
Seita.3G147000.1	Seita.5G265200.1	0.6646	0.2722	0.4095
Seita.5G321300.1	Seita.5G265200.1	1.4938	0.5144	0.3443
Seita.3G179300.1	Seita.5G321300.1	0.7762	0.3115	0.4013

Table S2 Synonymous values of orthologous *ARF* gene pairs between *S.italica* - O.sativa and *S.italica* - Z.mays.

Orthologous gene pairs		Ks
Seita.1G077200	Os02g06910	0.5015
Seita.1G090900	Os02g04810	0.5124
Seita.1G195500	Os02g35140	0.543
Seita.1G241500	Os02g41800	0.7386
Seita.3G020000	Os04g57610	0.4437
Seita.3G028100	Os04g56850	0.5432
Seita.3G147000	Os05g48870	0.4367
Seita.3G179300	Os05g48870	1.8779
Seita.3G321500	Os11g32110	0.9468
Seita.3G394000	Os12g41950	0.4605
Seita.4G040100	Os06g09660	0.5139
Seita.4G240600	Os06g48950	0.5799
Seita.4G257800	Os06g47150	0.5964
Seita.4G262300	Os06g46410	0.4842
Seita.5G004300	Os01g13520	0.5335
Seita.5G265200	Os01g48060	0.3888
Seita.5G321300	Os01g54990	0.4706
Seita.5G438100	Os11g32110	2.0716
Seita.6G212000	Os08g40900	0.4893
Seita.7G108100	Os04g36054	0.4964
Seita.8G135700	Os11g32110	0.5162
Seita.1G077200	Zm00001d053819	0.3389
Seita.1G090900	Zm00001d015243	0.2615
Seita.1G195500	Zm00001d016838	0.3171
Seita.1G241500	Zm00001d017284	0.6444
Seita.3G003300	Zm00001d002929	0.4236
Seita.3G020000	Zm00001d026590	0.247
Seita.3G028100	Zm00001d026540	0.3965
Seita.3G147000	Zm00001d043922	0.6088
Seita.3G179300	Zm00001d038698	0.3169
Seita.3G321500	Zm00001d041056	0.2785
Seita.3G394000	Zm00001d041497	0.2595
Seita.4G040100	Zm00001d045026	0.2672
Seita.4G240600	Zm00001d014690	0.2717
Seita.4G257800	Zm00001d014507	0.9827
Seita.4G262300	Zm00001d014377	0.3661
Seita.5G004300	Zm00001d039267	0.406
Seita.5G265200	Zm00001d043922	0.2237
Seita.5G321300	Zm00001d043431	0.2343

Seita.5G438100	Zm00001d011953	0.4063
Seita.6G212000	Zm00001d031522	0.3183
Seita.7G108100	Zm00001d003601	0.2192
Seita.7G169600	Zm00001d017284	1.5363
Seita.8G135700	Zm00001d049295	0.3847
Seita.9G219800	Zm00001d032683	0.4262

Table S3 Primers used in the qRT-PCR assay.

Gene name	F(5'-3')	R(5'-3')
Seita.1G077200	TCAACCAAAGCGGCATC	CTGTTTGCCCGACGAA
Seita.1G090900	CAGCCAACAATAGCCCAT	TCATCCGAAATCGCATTCCT
Seita.1G195500	TACAGATCAGCAGCTTGACC	TTGAGCGTAAACTTCATCGG
Seita.1G241500	ACCAGCGTCTACTACTTCC	TTCTCCTGCTCGTCGTCC
Seita.3G003300	GATTTCTGACACAATGGCAC	AACCCAACAGTATAGCCATG
Seita.3G020000	TAGCCCAACGTCATTACCAA	TCCACACATTTCGAGCTTC
Seita.3G028100	GTCGCCATTTGTAATTCCT	TCACTAATTCCCACGACT
Seita.3G147000	TTAGCGAAGCTGACCCTA	TAGTCAGCTCGATCTCCC
Seita.3G179300	GTCAACCACGTAGGCATC	TAGTTCGCCATCATTACCTC
Seita.3G321500	TGACATGCCAACTGTAACCA	TTGTGCCCTTCCTTGACTG
Seita.3G394000	CAAACTCACACTGGCGTTC	GTTGTCAATGGCTCAATCTCC
Seita.4G040100	TTGAGCCAGTTGCTACACC	GCAAAGTCATCACCAAGCCA
Seita.4G240600	ACAGAGTTTGTTGTACCGTT	ATGCCAGTTATTGTACCCAT
Seita.4G257800	GCGTTCCATTGTACCCAG	CCATCCGGGAAATAGCAC
Seita.4G262300	GCCATGCAAAATACGCTGA	TCCACTGCAACTCGCTTC
Seita.5G004300	GCACCTTCTGACTACTGGA	CAAGGCGTCTCACTCCGA
Seita.5G265200	TATCTATCGAGGCCAACCAC	GACGCACTCCTAATCGAAG
Seita.5G321300	TTAAGAGCCTCAACCGTCCA	ATCAACTTCACTAATGCCAAC
Seita.5G438100	CACATCTTTCGTGGGCAAC	ATAGCCCGCCTAACACCAA
Seita.6G212000	ACACTATTCAGTACCCGACA	CAAAGAGCGAGTACCCAT
Seita.7G108100	AGTTGAACTCGGATAAGCTC	CAACAGCCATTCCTTGCAT
Seita.7G169600	TTCGACAACGCTGACCTG	CACTCCGTATTGTGATCCTC
Seita.8G135700	TATTCAGAGGTCAGCCACGAA	CCTAACTCCAACACGCAAC
Seita.9G219800	CCTATCCTTACCGAGCAG	ATGCAAATAGATGAACCGGAA
Actin 2	GGAATTCAATAAGCACGACAG	TTGTTGTAAATCTCCGGGTT
